# Interventions to improve appropriateness of laboratory testing in the intensive care unit: a narrative review

**DOI:** 10.1186/s13613-024-01244-y

**Published:** 2024-01-15

**Authors:** Luigi Devis, Emilie Catry, Patrick M. Honore, Alexandre Mansour, Giuseppe Lippi, François Mullier, Mélanie Closset

**Affiliations:** 1 Department of Laboratory Medicine, Biochemistry, CHU UCL Namur, Université catholique de Louvain, Yvoir, Belgium; 2 Department of Laboratory Medicine, Hematology, CHU UCL Namur, Université catholique de Louvain, Yvoir, Belgium; 3 Department of Intensive Care, CHU UCL Namur, Université catholique de Louvain, Yvoir, Belgium; 4 Namur Thrombosis and Hemostasis Center (NTHC), Namur Research Institute for Life Sciences (NARILIS), Namur, Belgium; 5 Institute for Experimental and Clinical Research (IREC), Pôle Mont Godinne (MONT), UCLouvain, Yvoir, Belgium; 6 Department of Anesthesia and Critical Care, Pontchaillou University Hospital of Rennes, Rennes, France; 7 IRSET-INSERM-1085, Univ Rennes, Rennes, France; 8 Section of Clinical Biochemistry and School of Medicine, University Hospital of Verona, Verona, Italy

**Keywords:** Overuse, Inappropriate, Tests, Intensive care unit, Prescription, Minimum retesting interval, Interventions, Artificial intelligence

## Abstract

Healthcare expenses are increasing, as is the utilization of laboratory resources. Despite this, between 20% and 40% of requested tests are deemed inappropriate. Improper use of laboratory resources leads to unwanted consequences such as hospital-acquired anemia, infections, increased costs, staff workload and patient stress and discomfort. The most unfavorable consequences result from unnecessary follow-up tests and treatments (overuse) and missed or delayed diagnoses (underuse). In this context, several interventions have been carried out to improve the appropriateness of laboratory testing. To date, there have been few published assessments of interventions specific to the intensive care unit. We reviewed the literature for interventions implemented in the ICU to improve the appropriateness of laboratory testing. We searched literature from 2008 to 2023 in PubMed, Embase, Scopus, and Google Scholar databases between April and June 2023. Five intervention categories were identified: education and guidance (E&G), audit and feedback, gatekeeping, computerized physician order entry (including reshaping of ordering panels), and multifaceted interventions (MFI). We included a sixth category exploring the potential role of artificial intelligence and machine learning (AI/ML)-based assisting tools in such interventions. E&G-based interventions and MFI are the most frequently used approaches. MFI is the most effective type of intervention, and shows the strongest persistence of effect over time. AI/ML-based tools may offer valuable assistance to the improvement of appropriate laboratory testing in the near future. Patient safety outcomes are not impaired by interventions to reduce inappropriate testing. The literature focuses mainly on reducing overuse of laboratory tests, with only one intervention mentioning underuse. We highlight an overall poor quality of methodological design and reporting and argue for standardization of intervention methods. Collaboration between clinicians and laboratory staff is key to improve appropriate laboratory utilization. This article offers practical guidance for optimizing the effectiveness of an intervention protocol designed to limit inappropriate use of laboratory resources.

**Supplementary Information:**

The online version contains supplementary material available at 10.1186/s13613-024-01244-y.

## Background

Healthcare spending is increasing in the US and Europe, faster than economic growth [1, 2]. The use of clinical laboratory tests has also increased, due in part to greater accessibility and affordability [3, 4]. Despite accounting for a small proportion of healthcare expenses, clinical laboratories are involved in the majority of medical decisions, making them central players in healthcare [5–7]. However, there is indication of inappropriate use of laboratory resources, with 20% to 40% of overall tests deemed inappropriate [8, 9], and with estimates as high as 60% of coagulation tests and 70% of chemistry tests considered of doubtful clinical significance [10]. Overuse can cause hospital acquired-anemia and subsequent need for transfusion, increased costs, staff overload, patient discomfort and stress, incidental findings, additional unnecessary interventions, and infections (e.g., central line-associated bloodstream infection), whereas underuse can lead to missed or delayed diagnosis [8, 10–16]. Several techniques can reduce the volume of blood drawn for laboratory testing. These include utilizing small-volume tubes [17, 18], non-invasive measures, and residual blood from previous samples [19]. In a wider context, interventions could be conducted to improve the appropriateness of laboratory testing and ordering.

Multiple reviews have assessed the published literature of interventions led in non-intensive care units (ICU) wards and among primary care physicians [3, 4, 11, 20–33]. However, there are few published assessments of ICU-specific interventions to date. Of note, two systematic reviews have been previously published. The first, from Foster et al*.* [34], reviewed audit and feedback interventions to improve laboratory test and transfusion practice from inception to 2016, but did not evaluate other types of interventions. The second, from Hooper et al*.* [35], evaluated safety and efficacy of routine diagnostic tests (including mixed laboratory tests and radiographs ordering data) reduction in the ICU between 1993 and 2018, with a subsequent meta-analysis of costs savings. Based on our knowledge, there are no other reviews focusing on laboratory tests in the ICU for all types of interventions. Furthermore, artificial intelligence and machine learning (AI/ML) assisting tools are destined to be increasingly used in laboratory medicine [36–38]. Mrazek et al*.* [4] reviewed several AI-centered studies of relevance for laboratory medicine as they call it “the next logical step” in the pursuit of appropriateness. No review has explored so far the role of AI/ML-based solutions in interventions to improve the appropriateness of laboratory use in the ICU to the best of our knowledge.

We hence decided to review the available literature on interventions to reduce inappropriate testing in the ICU. As well as evaluating their effectiveness and costs savings, we try here to provide an assessment of their feasibility and persistence over time. The complete methodology for this review can be found in Additional file 1.

## Interventions to improve laboratory testing appropriateness in the ICU

### Education and guidance

Education and/or guidance (E&G) is one of the most common approaches used to limit the number of inappropriate tests in ICU (Table 1) as in non-ICU wards [4, 11, 20, 32, 33, 39]. E&G account for more than 40% of all interventions, with evidence of good effectiveness [23]. This strategy has long been used to regulate prescription of laboratory tests [24], as well as in more recent ICU interventions [40–51], even if E&G is often associated with other strategies (Table 1).

**Table 1:** Methodological details and results of studies conducted in intensive care units to reduce inappropriate use of laboratory tests

Article	Réfs.	Years	Study design (Article type)	Setting	Test(s) studied	Intervention(s) type(s)	Time periods	Test(s) reduction or outcome(s)	Costs savings estimate	Safety outcome(s)
Adhikari et al.	[57]	2022	Prospective BAA	N/S	FBC; BMP (*n.o.s.*)	Education (indications for testing, utilization of results, formal sessions, pamphlets, and flyers)	Pre-I: 2 mo Post-I: 2 mo	Increase in clinically indicated BMP of 19%; no statistical significance for FBC	N/S	N/S
Aloisio et al.	[81]	2019	Retrospective BAA (C.A.)	*n* = 366 patients	PCT	CPOE (prompts)	Pre-I: 9 mo Post-I: 9 mo	10% total reduction	EUR 6000 (/9 mo.)	N/S
Blum et al.	[40]	2015	Retrospective BAA	16-bed; *n* = 300 patients (159 pre-I; 141 post-I)	ABG	Guidance (literature search)	Pre-I: 3 mo Per-I: 9 mo Post-I: 3 mo	Mean reduction from 5.5 to 3.7 (−38%) ABG tests per patient	N/S	Decrease in MV and LOS
Bosque et al.	[136]	2019	Retrospective BAA (C.A.)	16-bed; *n* = 229 PD	N/S	Education (prices information via emails); A&F (number of tests ordered via emails)	Pre-I: 10 mo Per-I: 3 mo Post-I: 9 days	39% reduction in inappropriate tests for critical patients; no statistical significance for semi-critical patients	N/S	N/S
Cahill et al.	[41]	2018	Retrospective BAA (C.A.)	N/S	N/S	Education (iatrogenic anemia focus culture); Guidance (locally established)	Pre-I: 11 mo Post-I: 11 mo	23% reduction in laboratory orders; 21% reduction in blood specimens; 23% reduction in POCT specimens	N/S	No increase in LOS nor transfusion need
Castellanos et al.	[84]	2018	Prospective ITS	25-bed	PCT	CPOE (clinical decision support system implementation)	Pre-I: 4 mo Per-I: 4 consecutives periods of 28 days (ON1-OFF1-ON2-OFF2) Post-I: 28 days	0.807 TPD on Pre-I (= baseline), 0.662 (−18%) on ON1, 0.733 (−10%/baseline) on OFF1, 0.803 (−0.4%/baseline) on ON2, 0.792 (−2%/baseline) on OFF2, 0.807 (+ 0%/baseline) in Post-I	EUR 15000 (/y) if persistence of scenario “ON1”	N/S
Cismondi et al.	[98]	2012	Database	MIMIC-II database version 2.6; *n* = 40,426 patients	HCT, HB, PLT, CA, LACT, aPTT, INR/PT, FIB	AI (TS fuzzy modeling; inputs: heart rate, respiratory rate, oxygen saturation, temperature, arterial blood pressure, urine output, intravenous infusions volumes and packed red blood cells, fresh frozen plasma, and platelets transfusions)	N/A	Reduction in 50% of total amount of tests; 11.5% false negatives (= tests that would not be done following algorithm but were in fact appropriate)	N/S	N/A
Clouzeau et al.	[86]	2019	Controlled trial	2 × 12-bed; *n* = 5707 patients (3315 interventions; 2392 controls)	CHEM (CREAT, BUN, K, CA, GLU, TBIL, NA, PROT, P, CRP, BNP, PCT, ALT, AST, GGT, TROP); FBC; COAG (*n.o.s.*), FIB	Education (prices information, formal sessions); A&F (review of tests requested); Gatekeeping (regulation of CA, P, PROT, CREAT, LFT, ABG, TROP testing, proscribe systematic daily electrolytes testing, CRP, systematic check of a first normal electrolyte test)	Pre-I: 1 y Per-I: 1 y. with supervision and 1 y. without supervision Post-I: 7 mo	Per-I (with supervision): −59% total tests/baseline; Per-I (without supervision): −48% total tests/baseline; Post-I: −30%/baseline	EUR 500000 (/y.)	No adverse outcome. No increase in mortality
Conroy et al.	[80]	2021	Prospective BAA	24-bed	BMP, MG, P, iCA, FBC, COAG (PT, aPTT, INR/PT), TG, CREAT, NH3, pH	Education (posters, new comers, reminders on round checklist); A&F (advice from other clinicians); Gatekeeping (withdrawal of routine admission penal testing); CPOE (warnings minimal retesting interval not respected)	Pre-I: 2 y Per-I: 9 mo	20% reduction per PD	USD 30000 (/week)	No increase in mortality nor morbidity (RRT, CLABSI, STATs)
de Bie et al.	[70]	2016	Prospective BAA	48-bed; 9000 PD/y	FBC; COAG (PT/INR, aPTT); CHEM (CREAT, BUN, CA, CL, P, ALB, CRP, AST, ALT, TBIL, ALP, AMY); CARD (CK, CK-MB, TROP)	Education (lectures); Gatekeeping (change in daily morning and post-cardiac surgery routine panels; withdrawal of weekly routine panel constituted of AST, ALT, ALP, AMY, TBIL) CPOE (change in POCT devices presets)	Pre-I: 15 mo Per-I: 15 mo	24% reduction in total testing	N/S	N/S
Della-Volpe et al.	[42]	2014	Prospective BAA	71-bed; 22530 ABG/y	ABG	Guidance (locally established following literature review); Education (indications for testing; via email and posters, educational sessions)	Pre-I: 6 weeks Per-I: 6 weeks	Reduction from 35.3 to 26.5 ABGs per 100 PD	USD 87565 (/y.)	N/S
Dhanani et al.	[74]	2018	Prospective BAA	22-bed; *n* = 3250 patients (1141 pre-I; 1067 Per-I; 1042 Post-I)	FBC; CHEM (BUN, CREAT, electrolytes, LFT, CA, MG, P); COAG (INR/PT, aPTT, FIB)	Education (emails, posters, weekly staff meetings, scheduled education session, prices information); Guidance (locally established following literature review); CPOE (redesign of request form); A&F (internal audit, daily feedback on tests ordered)	Per-I: 6 mo Per-I: 6 mo Post-I: 6 mo	Per-I: 28% total (FBC 12%, CHEM-20 44%, COAG 70%); Post-I: 26% total	Per-I: reduction of USD 28 (/PD); Post-I: reduction of USD 24 per PD	No increase in mortality nor LOS; no increase in complications (Hb level, MV)
Dodek et al.	[76]	2018	Prospective BAA (C.A.)	15-bed	MG	Education (new comers to the service, posters); CPOE (prompts); Guidance (locally established following literature review)	Pre-I: 12 mo Post-I: 5 mo	24% reduction in routine tests with stable non-routine testing	N/S	No increase in mortality nor LOS
Fresco et al.	[43]	2016	Prospective BAA (C.A.)	*n* = 606 patients (274 pre-I; 342 post-I)	N/S	Guidance (at patient bedside)	Pre-I: 6 mo Post-I: 6 mo	27% reduction in routine testing	EUR 124000 (/6 mo.) + EUR 53000 (/6 mo.) in transfusion economy	27% reduction in blood transfusion; no increase in mortality; no difference in nosocomial infections
Goddard & Austin	[44]	2011	Prospective BAA (C.A.)	6-bed	FBC; CHEM (BUN, electrolytes, LFT, CA, P, ALB, CRP); COAG (*n.o.s.*)	A&F (audit of personnel via order chart)	Pre-I: 100 days Post-I: 100 days	33% total reduction (COAG 52%, LFT 54%, BP 54%)	GBP ~ 3000 per bed	N/S
Gray & Baldwin	[137]	2014	Prospective BAA (C.A.)	26-bed	FBC; CHEM (BUN, electrolytes, LFT, CRP, BP, MG); COAG (*n.o.s.*)	Education (*n.o.s.*)	Post-I: 28 days	N/S	EUR ~ 38,000 (/y.)	N/S
Hall et al.	[138]	2016	Prospective BAA (C.A.)	20-bed; *n* = 10 patients	N/S	Guidance (clarification of precedent implemented guidelines); Education (posters at patient bedside)	Per-I: 6 random days over a 2 mo. period	Reduction from 46 to 41% in inappropriate tests	N/S	N/S
Han et al.	[139]	2014	Prospective BAA	600-bed academic hospital (*n.o.s.*)	iCA, CA, CL, MG, P	Financial incentive; Guidance (locally established)	Pre-I: 1 y Post-I: 1 y	47% total reduction similarly distributed across all tests	USD 74000 (/y.) and USD 1.7 million in billable charges	No change in quality metrics
Iosfina et al.	[79]	2013	Prospective BAA (C.A.)	15-bed	FBC, BUN, electrolytes, CREAT	Education (new comers, periodical meetings with clinicians, reminders on checklist); Guidance (locally established); CPOE (prompt with indications for testing)	Pre-I: 1 y Post-I: 3 mo	24% total reduction	USD 22000 in direct costs	No increase in STAT labs
Jefferson & King	[68]	2018	Prospective BAA	16-bed; *n* = 81 patients (41 Pre-I; 40 Post-I)	FBC, CREAT, HCT, LACT, P, PT, TBIL	Education (reminders on checklist, reminders on computers and at bedside); A&F (presence of PI during multidisciplinary rounds to discuss next 24 h tests requests)	Pre-I: 2 weeks Per-I: 2 weeks	NSS	NSS	No statistical differences in mortality and morbidity (reintubation within 48 h, hemorrhage, cardiac arrest, dysrhythmia, hypotension)
Khan et al.	[75]	2019	Prospective BAA (C.A.)	24-bed	N/S	Education (awareness, visual reminders); CPOE (modification of EMR notes template); A&F (periodical feedback cycles during intervention)	N/S	Reduction in tests from 9.4 to 7.5 (-20%) TPD; increase in discussed clinical cases for testing in morning round from 30 to 95%	N/S	N/S
Kotecha et al.	[46]	2017	Prospective BAA	12-bed; *n* = 207 patients (103 Pre-I; 104 Post-I)	COAG (*n.o.s.*); LFT (*n.o.s.*), MG, P, LACT, TROP	A&F (audit of testing practice, interview of clinicians); Guidance (locally established); Education (*n.o.s.*)	Data collected at 2 mo. Post-I; Persistence assessed 1 y. Post-I	Overall reduction of 22% of inappropriate tests; reduction of 39% for LFT testing, 36% COAG, 53% MG, 62% P, 14% CARD, 12% LACT Persistence (/baseline): reduction of 21% for MG, 46% P, 30% LFT, 37% COAG	N/S	No increase in delay of procedures or in cardiac arrhythmias
Kumwilaisak et al.	[47]	2008	Prospective BAA	25-bed; *n* = 1117 patients (558 Pre-I; 559 Post-I)	COAG (*n.o.s.*); CHEM (*n.o.s.*), GLU, CARD (*n.o.s.*); ABG	Education (monthly sessions, flyers, emails); Guidance (locally established following literature review); A&F (monthly email on the outcome of the project)	Pre-I: 6 mo Post-I: 6 mo Persistence assessed 1 y. Post-I	38% reduction on total testing; + 178% increase in tests with clear and appropriate indication; reduction from 21 to 16 (−24%) TPD; decrease in testing of GLU 51%, ABG 48%, CHEM 37%, COG 30%, CARD 23%	N/S	No increase in number of critical values, transfusion need, mortality, LOS, MV, re-admission
Lee & Maslove	[93]	2015	Database	MIMIC-II database version 3.2.09; *n* = 29,149 patients	HCT, PLT, WBC, GLU, HCO3, K, NA, CL, BUN, CREAT, LACT	Information theory (reviewed in AI-based interventions)	N/A	CREAT-BUN, HCO3-LACT and NA-CL pairs contain a great degree of redundancy of information; PLT, BUN and CREAT are the most redundant tests performed on days 2 and 3 of ICU stay	N/S	N/A
Leydier et al.	[48]	2016	Retrospective BAA (C.A.)	*n* = 3568 patients	N/S	Guidance (*n.o.s.*)	Post-I: 5 years	Reduction from 18.1 to 6.4 TPD (−65%) after 5 y	EUR 318000 in 2012 on 5 most expensive tests (*n.o.s.*)	No increase in transfusion, HB levels, or mortality
Litton et al.	[87]	2020	Prospective BAA	30-bed; *n* = 5102 patients (2477 Pre-I; 2625 Post-I)	FBC; CHEM (BUN, electrolytes, MG, CA, LFT (*n.o.s.*), ABG, CRP, PCT); COAG (INR/PT, aPTT); TROP	Education (relevance of tests); Guidance (*n.o.s.*); Gatekeeping (withdrawal of routine panel testing and routine admission panel testing); A&F (number of tests during intervention);	Pre-I: 12 mo Post-I: 12 mo	Reduction of routine admission tests from 47.0 to 24.9 (−47%) per ICU admission (= ~ 50,000 in absolute number of tests /y.)	AUD 794000 /y	No increase in mortality nor LOS; reduction of transfusions and MV needed
Lo et al.	[77]	2020	Prospective BAA (L.Ed.)	15-bed	MG	Education (monthly sessions); CPOE (prompts); Guidance (indications for testing)	Pre-I: 1 y Post-I: 11 mo	Overall reduction from 0.71 to 0.57 (−20%) TPD with reduction from 0.57 to 0.41 (−39%) for routine tests and stable non-routine tests	CAD 4500 /y	No difference in mortality or LOS
Maguet et al.	[58]	2015	Prospective BAA (L.Ed.)	65-bed; *n* = 1817 patients (886 Pre-I; 931 Per-I)	FBC; CHEM (*n.o.s.*); ABG; COAG (PT, aPTT)	Education (daily information, reminders at bedside, prices information)	Pre-I: 4 mo Per-I: 4 mo	Reduction of 7.5% per patient-day	*N/A* (costs of labs and radiographs mixed)	N/S
Martinez-B. et al.	[88]	2017	Prospective BAA	98-bed	ABG	Education (classic sessions, posters, stickers on POCT devices, monthly emails); Guidance (locally established following literature review); A&F (audit prior to intervention; monthly feedback emails)	Pre-I: 1 y Post-I: 1 y	43%	USD 98500 (/y.)	No difference in APACHE, LOS, mortality, MV, re-admission
Merkeley et al.	[78]	2016	Prospective BAA	15-bed; *n* = 1440 patients (709 Pre-I; 731 Post-I)	FBC; electrolytes/renal panel	Guidance (locally established following literature review); Education (reminders on morning round checklist, posters, formal sessions); CPOE (prompt on accepted indications)	Pre-I: 1y Post-I: 1 y	Reduction in routine FBC testing from 0.97 to 0.83 TPD (−14%); increase in non-routine FBC testing from 0.37 to 0.40 TPD (+ 8%); Reduction in routine electrolytes testing from 0.96 to 0.83 TPD (−13%); increase in non-routine electrolytes testing from 0.32 to 0.34 (+ 6%)	CAD 11000 (/y.)	No increase in mortality nor complications (number of STAT labs, transfusion, LOS)
Musca et al.	[90]	2016	Controlled trial	23-bed; *n* = 253 patients (100 Pre-I; 153 Post-I)	COAG (INR/PT, aPTT, FIB); CHEM (BUN, CREAT, electrolytes) for control	Education (face-to-face, posters, email, electronic material, prices information); Guidance (locally established); A&F (feedback mid-study via email)	Pre-I: 3 mo Post-I: 2 mo	64% (vs. 15% for control tests)	60% of total costs (AUD 98000 /y.); AUD ~ 3,8 million to AUS/NZL scale a year	No Increase in complications (bleeding, morbidity)
Prat et al.	[49]	2009	Retrospective BAA	15-bed; *n* = 1175 patients (541 Pre-I; 634 Post-I)	COAG (PT, aPTT, FIB); FBC; CHEM (electrolytes, BUN, CREAT, GLU, PROT, CA, P, TBIL, ALP, GGT, AST/ALT, TROP, CK, LACT); ABG	Guidance (locally established following literature review); Education (sessions); A&F (monthly feedback on number of tests)	Pre-I: 1 y Post-I: 1 y	Relative reduction from 38% to 71.5% (overall reduction of ~ 50% of routine tests)	*N/A* (costs of labs and radiographs mixed)	No difference in mortality nor morbidity (SAPS, MV); decrease in LOS
Raad et al.	[85]	2017	Prospective BAA	18-bed	N/S	Education (flyers, emails, monthly sessions for resident, during rounds); Guidance (locally established; on urgency of tests); Gatekeeping (withdrawal of routine testing, 24 h max. anticipation for blood analysis prescribing, 1 unique draw at 2 p.m. for tests requested at morning round, withdrawal of ionogram testing on POCT devices); A&F (daily feedback on tests ordered);	Pre-I: 3 mo Post-I: 9 mo	Reduction from 39.4 to 26.9 (−32%) TPD; reduction of patients having daily blood draw from 100 to 12%; reduction of POCT testing from 7.3 to 1.2 (−83%)TPD	*N/A* (costs of labs and radiographs mixed)	No increase in mortality nor LOS; stable CLABSI
Rachakonda et al.	[69]	2017	Prospective BAA	*n* ~ 230 patients/mo	CHEM (electrolytes, LFT, CA, P, MG, AMY, LIP, CREAT, BUN); FBC; COAG (*n.o.s.*); ABG; CARD (CK, CK-MB, TROP); microbiological cultures, microbiological screening swabs, therapeutic drugs level	Education (monthly sessions); A&F (review of tests requested; monthly feedback on the results of the study)	Pre-I: 6 mo Post-I: 6 mo	N/S	Overall reduction in laboratory costs of 12%	No difference in mortality, LOS, nor severity (APACHE III)
Shen et al.	[50]	2019	Controlled trial	25-bed (46-bed for control)	FBC with differential	A&F (audit of practices and comparison with other hospitals of the same network); Guidance (locally established); Education (*n.o.s.*)	Pre-I: 2 weeks (2 mo. before intervention) Per-I: 2 weeks Post-I: 2 weeks (2 mo. after intervention)	Reduction in total FBC w/diff Per-I (−20% vs. *NSS* control) and Post-I (−19% vs. −19% control); reduction in repeated (within 22 h) FBC w/diff Per-I (−31% vs. −21% control) and Post-I (−27% vs. −32% control)	N/S	No negative impact nor delay in the diagnosis of sepsis, no change in management plan
Simvoulidis et al.	[140]	2020	Retrospective BAA (C.A.)	*n* ~ 1300 patients	N/S	A strategy (*n.o.s.*)	1 y	Reduction in > 50% total requests	USD 150000 (/y.)	No negative impact (mortality, LOS, use of invasive resources)
Sugarman et al.	[71]	2020	Prospective BAA (C.A.)	16-bed; *n* = 191 PD	FBC, electrolytes, BUN, LFT, CRP, MG, P, COAG (*n.o.s.*)	Gatekeeping (self-limitation to 25% of requests without clear clinical indication maximum)	Per-I: 4 weeks	Rate of tests requested without clear clinical indication < 25%: CRP (13.1%), FBC (15.4%), BUN & electrolytes (18.1%) Rate of tests requested without clear clinical indication > 25%: LFT (51%), MG (42.2%), P (42.7%), COAG (40.4%)	25% of costs deemed inappropriate	N/S
Thakkar et al.	[61]	2015	Prospective BAA	400-bed; *n* = 1970	CBC, BMP, CMP, PT/INR, PTT	Educational sessions, flyers, weekly emails with following message: “(1) question the utility of every blood test and order the tests only if the result will affect patient care, (2) think about the sizable impact that costs of blood tests have on health care expenditures, and (3) consider “adding on” tests to blood samples that have already been collected whenever possible”	Pre-I: 2 mo Post-I: 2 mo	Total tests decreased from 13742 pre-I to 13528 post-I (2%)	USD 6.33 per PD	N/S
Tyrrell et al.	[72]	2015	Prospective BAA	33-bed	BUN, CREAT, electrolytes, BP (CA, ALB, ALP, PROT, calculated globulin), LFT, CA, ALB, MG, P, CRP, FBC, COAG (*n.o.s.*)	Gatekeeping (implementation of MRI [72 h for BP and LFT; 24 h for CRP] then replaced by SRPT [3 blood draws per week]); Education (trainee medical and nursing staff)	Pre-I: 6 mo Per-I (MRI): 2 periods of 6 mo Per-I (SRPT): 2 periods of 6 mo	22% total reduction after MRI introduction; 33% total reduction compared to baseline after SRPT introduction	N/S	N/S
Vezzani et al.	[51]	2013	Prospective BAA (L.Ed.)	N/S	CHEM (*n.o.s.*)	A&F (audit prior to intervention); Education (*n.o.s.*); Guidance (locally established following literature review)	Pre-I: 1 mo Post-I: 2 mo. separated by 7 mo	71% decrease in routine testing and 29% in non-routine testing	37% decrease in costs for routine testing and 63% of costs in non-routine testing	No difference in mortality rate, LOS, nor severity (SAPS II)
Viau-Lapointe et al.	[91]	2018	Prospective BAA (C.A.)	N/S	LFT (*n.o.s.*); COAG (*n.o.s.*)	A&F (audit prior to intervention via interview and electronic form); Education (sessions and posters); Guidance (locally established); Gatekeeping (removal of tests deemed unnecessary)	N/S	Reduction for LFT from 0.65 to 0.25 TPD (−61%); *NSS* for COAG	N/S	N/S
Walsh et al.	[89]	2020	Prospective BAA (C.A.)	58-bed	ABG	Education (*n.o.s.*); Guidance (*n.o.s.*)	Pre-I: 6 mo Post-I: 6 mo	31% total reduction	AUD 750000 (/y.)	No difference in mortality nor severity (APACHE III)
Yorkgitis et al.	[60]	2018	Controlled trial	18-bed; *n* = 307 patients (152 intervention; 155 control)	COAG (PT, aPTT); FBC; CHEM (*n.o.s.*); ABG	Education (a reminder was added in the checklist round: “What laboratory tests are medically necessary for tomorrow?”; posters)	Pre-I: 3 mo Per-I: 3 mo	NSS	N/A	No difference in mortality, LOS, severity nor morbidity
Yu et al.	[102]	2020	Database	MIMIC-III database; *n* = 38,773 patients	NA, K, CL, HCO3, CA, MG, P, BUN, CREAT, HB, PLT, WBC	AI (same 2 double-layer Long Short Term Memory Networks modeling as Yu et al*.*[101] with self-feeding and a corruption strategy; the model had 4 tasks: 1° predict normal vs anormal, 2° predict transition from normal to abnormal or vice-versa, 3° predict numeric value, 4° predict appropriateness; inputs: vitals (*n.o.s.*), time differences from the last record, race, gender)	N/A	Omissions of 20% of total tests with 98% accuracy (2% false negatives) of abnormality predictions of the omitted tests	N/S	N/A
Yu et al.	[101]	2020	Database	MIMIC-III database; *n* = 41,113 patients	NA, K, CL, HCO3, CA, MG, P, BUN, CREAT, HB, PLT, WBC	AI (2 double-layer Long Short Term Memory Networks modeling with self-feeding strategy)	N/A	33% reduction at > 90% accuracy (< 10% false negatives); 15% reduction at > 95% accuracy	N/S	N/A

*A&F* audit and feedback, *ABG* arterial blood gas, *ALB* albumin, *ALP* alkaline phosphatase, *ALT* alanine aminotransferase, *APACHE* Acute Physiology and Chronic Health Evaluation score, *aPTT* activated partial thromboplastin time, *AST* aspartate aminotransferase, *AUS* Australia, *BAA* before and after study, *BMP* basic metabolic panel, *BNP* brain natriuretic peptide, *BP* bone profile, *BUN* blood urea nitrogen, *C.A.* conference abstract, *CA* calcium, *CARD* cardiac enzymes, *CK* creatine kinase, *CK-MB* creatine kinase myocardial band, *CL* chloride, *CLABSI* central line-associated bloodstream infection, *CO2* carbon dioxide, *COAG* coagulation tests, *CPOE* computerized physician order entry, *CREAT* creatinine, *CRP* C-reactive protein, *EMR* electronic medical record, *FBC* full blood count, *FIB* fibrinogen, *GGT* gamma-glutamyltransferase, *GLU* blood glucose, *HB* hemoglobin, *HCT* hematocrit, *iCA* ionized calcium, *INR* international normalized ratio, *ITS* interrupted time series, *K* potassium, *LACT* lactate, *LEd* letter to the editor, *LFT* liver function tests, *LOS* length of stay, *MG* magnesium, *mo.* Months, *MV* mechanical ventilation, *N/A* not applicable, *N/S* not specified in the study, *NA* sodium, *NH3* ammonium, n.o.s. not otherwise specified, *NSS* not statistically significant, *NZL* New Zealand, *P* phosphate, *PCT* procalcitonin, *PD* patient-days, *Per-I* per-intervention, *POCT* point-of-care testing, *Post-I* post-intervention, *Pre-I* pre-intervention, *PROT* protides, *PT* prothrombin time, *RRT* renal replacement therapy, *SAPS* Simplified Acute Physiology Score, *SRPT* scheduled routine panel testing, *STATs* STAT laboratory tests, *TBIL* total bilirubin, *TG* triglycerides, *TPD* test/patient/day, *TROP* troponin, *y.* years

Education can take various forms: formal sessions, staff meetings, peer group discussions, emails, flyers, posters, bedside reminders, content in the intranet, educational content on electronic devices such as tablets, etc*.* The fundamental purpose of an educational approach is to raise awareness of the need to change practice change towards more appropriate use of laboratory resources [52]. Educational strategies are frequently used because they are relatively accessible and inexpensive, can reach many people at once and generally fit within the logical framework of the intervention—the intervention is often explicitly explained to clinicians.

In the broadest sense, guidance for laboratory testing includes advice for clinicians on selecting the "right test, at the right time, for the right patient" [53]. In recent years, guidance has increasingly considered the principle that “less is more” [14, 54, 55], aiming to limit inappropriate tests. In France, there are national ICU guidelines on appropriateness of requesting laboratory tests and chest radiographs [56]. Guidance are developed with the assistance of (local) experts [41, 44, 50], following literature review [46], or a combination thereof [40, 42, 49, 51], or sometimes in response to an internal quality improvement study [46] (Table 2). Few interventions have used a guidance-based strategy alone. E&G-based interventions are effective, depending on the test (Table 1). They may even have relatively permanent effect over time [46].

**Table 2:** Indications for testing used in guidance-based interventions

Article	Réfs.	Years	Tests concerned	Type of indications	Indications for testing
Blum et al.	[40]	2015	ABG	Guidance locally established	*Not otherwise specified*: re-evaluation of pre-existing indications for testing (change in ventilator settings, respiratory or cardiac event, routine testing, metabolic event, pre- and postintubation, pre- and postextubation, follow-up on abnormal test results, unreliable pulse oximetry data, altered mental status) based on “evidence-based review of the literature”
Cahill et al. (C.A.)	[41]	2018	Not otherwise specified	Not otherwise specified	Not otherwise specified
Della-Volpe et al.	[42]	2014	ABG	Guidance locally established	Exclusions: age < 18, acute stroke, VBG; Indications: 1° Hemodynamic instability; 2° Oxygenation (sat < 88% AND decrease > 5% from baseline); 3° Suspected metabolic acid/base abnormality; 4° Respiratory distress (with one of: -accessory muscle use, -altered mental status, -respiratory rate increase, -diaphoresis, -cyanosis); 5° Ventilation changes (change in MODE, change in PEEP, change in minute ventilation, change in FiO_2_, daily ABG, weaning trial ABG, postextubation ABG); 6° Post-op initial ABG; 7° Other
Dhanani et al.	[74]	2018	FBC; COAG (INR/PT, aPTT, FIB); CHEM (BUN, CREAT, electrolytes, LFT, CA, MG, P);	Guidance locally established	1° Daily testing: FBC, BUN, electrolytes; 2° Twice weekly: FBC (appears twice), CHEM 20; 3° Coagulation only as required (order individual tests); 4° Drug levels (*not otherwise specified*): reduce rate of monitoring if at stable levels
Fresco et al. (C.A.)	[43]	2016	Not otherwise specified	Not otherwise specified	Not otherwise specified
Hall et al. (C.A.)	[138]	2016	Not otherwise specified	Not otherwise specified	Not otherwise specified
Han et al.	[139]	2014	CA, CL, MG, P	Guidance locally established	1° CL: presence of acidosis on ABG, HCO3 < 20 mEq/L, to calculate presence of anion gap; 2° MG: clinical evidence of poor nutrition, prolonged non-*per os* (NPO) status, heavy diuresis; 3° P: clinical evidence of poor nutrition, prolonged non-*per os* (NPO) status; 4° CA: use of blood products outside perioperative setting, suspicion for multiple endocrinopathies
Iosfina et al. (C.A.)	[79]	2013	FBC; BUN, electrolytes, CREAT	Guidance locally established	Not otherwise specified
Kotecha et al.	[46]	2017	COAG (*n.o.s.*); LFT, MG, P, LACT, TROP	Guidance locally established	1° Always appropriate: BMP, FBC; 2° MG: volume loss, arrythmia, receiving repletion; 3° P: receiving repletion, malnutrition, diabetic ketoacidosis, hyperphosphatemia from renal disease; 4° LFT: abnormal liver function tests, liver disease or injury, hepatotoxic medication; 5° COAG: bleeding, coagulopathy, on anticoagulation, planned procedure; 6° LACT: sepsis, suspected mesenteric ischemia, trending initial elevated level; 7° TROP: myocardial injury, active ischemia
Kumwilaisak et al.	[47]	2008	ABG; CHEM (*n.o.s.*), GLU, CARD (*n.o.s.*); COAG (*n.o.s.*);	Guidance locally established	1° Routine daily laboratory tests include FBC, NA, K, CL, CO2, MG, P, BUN, CREAT, GLU; 2° ABG and COAG are not routine; 3° Biomarkers of myocardial injury include CK-MB at baseline, TROP T at baseline, 8 and 16 h; 4° Plans for laboratory testing are discussed at the time of each patient’s rounds; 5° All tests require a written order in the POE. In emergencies, nurses can send tests according to their best judgment; such tests are later discussed with the house officer and an order is entered at that time
Leydier et al. (C.A.)	[48]	2016	Not otherwise specified.	Not otherwise specified	Not otherwise specified
Litton et al.	[87]	2020	FBC; COAG (INR/PT, aPTT); CHEM (BUN, electrolytes, MG, CA, LFT, ABG, CRP, PCT, TROP)	Guidance locally established	*Not otherwise specified:* “The pre-intervention ICU guideline involved conducting a routine panel of diagnostic tests on admission to ICU in addition to scheduled daily (morning) tests, unless otherwise directed by the training team. Post-intervention, the guideline was changed for admission and daily testing, so that only diagnostic tests deemed clinically indicated and explicitly suggested by the treating ICU team were requested.”
Lo et al. (L.Ed.)	[77]	2020	MG	Guidance locally established	1° Suspected hypomagnesemia with plans to replete in the setting of renal failure; 2° Optional in suspected hypermagnesemia
Martinez-B. et al.	[88]	2017	ABG	Guidance locally established	1° Should an ABG be drawn ? ∟ Oxygenation → acute decompensation ? Yes = Draw ABG → intervention required ? Yes = Follow further interventions with SpO_2_ if it correlates ± 3% with SaO_2_ ∟ Ventilation → acute decompensation or change of minute ventilation ? Yes = Draw ABG → intervention required ? Yes = Follow further interventions with ABG ∟ Acid–base → new or worsening acid–base disorder suspected ? Yes = Draw ABG → interventions required ? Yes = Follow further interventions with ABG 2° Do not draw an ABG if a disorder is not suspected or an intervention is not required; 3° Do not draw an ABG for spontaneous breathing trial; 4° Follow pulse oximetry after planned changes of FiO_2_ or positive end-expiratory pressure; 5° Do not use venous blood gas as surrogates for ABG; 6° Consider removing arterial lines as soon as clinically indicated
Merkeley et al.	[78]	2016	FBC; Electrolytes/renal panel (*n.o.s.*)	Guidance locally established	1° FBC: suspected anemia, suspected bleeding, suspected infection, suspected leucopenia, suspected thrombocytopenia, other (to be specified by physician); 2° Electrolytes/renal panel: suspected new electrolyte abnormalities, documented electrolyte abnormalities that are being corrected, suspected or ongoing kidney injury, other (to be specified by physician)
Musca et al.	[90]	2016	COAG (INR/PT, aPTT, FIB);	Not otherwise specified	1° On ICU admission: order screening coagulation profile if not done that day; 2° Significant bleeding: order coagulation profile as required; 3° New thrombocytopenia (< 50), liver failure or DIC before significant procedure: order coagulation profile once and then daily if abnormal; 4° Warfarin therapy with isolated high INR (> 1.3): INR only, daily or less when patient improving; 5° Heparin therapy with isolated high aPTT (> 42 s): aPTT only, as per heparin protocol, or daily or less if patient improving; 6° Coagulation profile abnormal but none of the above: consider ordering coagulation profile second daily or less if patient improving
Prat et al.	[49]	2009	FBC; COAG (PT, aPTT, FIB, coagulation factors); CHEM (electrolytes, BUN, CREAT, GLU, PROT, CA, P, TBIL, ALP, GGT, AST/ALT, TROP, CK, LACT); ABG	Guidance locally established	1° FBC, PLT: upon admission in ICU only if not done in emergency ward or other hospital unit, during ICU stay once or two times a week (of if bleeding event is suspected); 2° PT, aPTT, FIB: upon admission in ICU when DIC or hepatic failure, during ICU stay once or two times a week (if heparin treatment once a day until aPTT ok and after 2–3 a week; 3° Coagulation factors (*not otherwise specified*): upon admission in ICU when DIC or hepatic failure, during ICU stay when vitamin K deficiency, DIC or suspected hepatic failure; 4° Electrolytes, BUN, CREAT: upon admission in ICU only if not done in emergency ward or other hospital unit, during ICU stay once a day if metabolic abnormalities (NA or K) or when renal failure and for other situations once or twice a week; 5° Urinary electrolytes: not upon admission in ICU except if severe hyponatremia, during ICU stay once a day when metabolic abnormalities or renal failure and in other situations once a week; 6° CA, P: upon admission in ICU when renal failure or denutrition, during ICU stay once a week or depending on clinical context (prolonged length of stay, parenteral nutrition, rhabdomyolysis); 7° ABG: not upon admission in ICU except if respiratory failure, during ICU stay one hour after intubation, once a day if pulmonary failure with FiO_2_ > 60%, once every two days if pulmonary failure with FiO_2_ < 60%, twice a week if no pulmonary failure; 8° TBIL, ALP, GGT, ALT/AST: upon admission in ICU if clinical context, during ICU stay if clinical context and once a week when parenteral nutrition or under mechanical ventilation; 9° TROP: if myocardial infarction suspected and when confirmed once a day until decrease; 10° CK: if rhabdomyolysis suspected and when confirmed once a day until level < 1500 IU/L; 11° LACT: in case of unexplained metabolic acidosis and two times a day in case of shock
Raad et al.	[85]	2017	Not specified	Guidance locally established	Not on indications for testing per se but on the urgency of tests requested: *not otherwise specified*
Shen et al.	[50]	2019	FBC with differential	Guidance locally established	(Trauma Burn ICU setting.) Fresh trauma ? → NO = Follow unit protocol → YES = Switch from FBC with diff. to FBC no diff. for every 4–6 h. If patient stable at 48 h, discontinue current FBC order and order FBC with diff. for every 12 h. If patient not stable at 48 h, continue order of FBC no diff. for every 4–6 h until stabilization
Vezzani et al. (L.Ed.)	[51]	2013	CHEM (*n.o.s.*)	Guidance locally established	Not on indications for testing per se but on how to enhance appropriateness of testing: 1° Use specific panel (*not otherwise specified*) of tests for patients’ admission testing; 2° Do not practice bundling of multiple laboratory tests; 3° Order non-routine tests only on suspicion of disease, do not search for abnormal values to be corrected; 4° Once a year, examine testing practice in order to point out excessive or inappropriate test ordering that might be target for actions
Viau-Lapointe et al. (C.A.)	[91]	2018	COAG (*n.o.s.*); LFT	Not otherwise specified	Not otherwise specified
Walsh et al. (C.A.)	[89]	2020	ABG	Guidance locally established	ABG testing is inappropriate if performed: 1° At regular (*not otherwise* specified) interval in stable patients; 2° At change of shift; 3° When taken concurrently with other blood tests; 4° In response to a decrease in ventilation or oxygen delivery; 5° After a treatment was ceased in a stable patient

*ABG* arterial blood gas, *ALP* alkaline phosphatase, *ALT* alanine aminotransferase, *APTT* activated partial thromboplastin time; *AST* aspartate aminotransferase, *BMP* basic metabolic panel, *BUN* blood urea nitrogen, *C.A.* conference abstract, *CA* calcium, *CARD *cardiac enzymes, *CHEM* biochemistry tests, *CK* creatine kinase, *CK-MB* creatine kinase myocardial band, *CL* chloride, *CO2* carbon dioxide, *COAG* coagulation tests, *CREAT* creatinine, *CRP* C-reactive protein, *DIC* disseminated intravascular coagulation, *FBC* full blood count, *FIB* fibrinogen, *GGT* gamma-glutamyltransferase, *GLU* blood glucose, *INR* international normalized ratio, *K* potassium, *LACT* lactate, *L.Ed.* letter to the editor, *LFT* liver function tests, *MG* magnesium, *NA* sodium, *P* phosphate, *PCT* procalcitonin, *PT* prothrombin time, *TBIL* total bilirubin, *TROP* troponin

Few studies have looked at education alone or as a main strategy [57–60]. Maguet et al*.* [58] achieved a sustained tests reduction of 7% per patient-day by providing daily information, indications for testing, prices information and reminders at the patient bedside. Similarly, Adhikari et al*.* [57] provided care staff with a feedback of an audit on prescription patterns, along with literature data, flyers, posters and formal education on appropriate prescription. Analyzing 153 records post-intervention, they reported an increase in appropriate prescription from 60 to 79% for full blood count (FBC). However, the effect was not statistically significant on basic metabolic panel (BMP) requests.

E&G strategies have several limitations. First, low-intensity education-based interventions are not effective enough to induce substantial change in prescribing behavior. Yorkgitis et al*.* [60] investigated the impact of a “gentle reminder” (i.e., the question “What laboratory tests are medically necessary for tomorrow?”) during morning round. The intervention had no significant effect on test reduction. Second, an important factor of success in education-based interventions is repetition, for example, weekly or daily [52, 58, 61]. This can prove difficult to maintain over time. A solution could involve the development of continuing education for young residents and rotating staff [20, 62], as required in ISO15189:2022 [63]. Third, there is significant heterogeneity in guidance and test(s) considered in the interventions we retrieved. The guidance was locally established and, as local behaviors vary widely between hospitals [64–66], practices also exhibit high variability between studies. Some guidelines focus on first-test indications, while others focus on retest indications. Some consider certain elements as always appropriate for routine testing, such as BMP and FBC [46], whereas others are tailored to a specific test [50] (Table 2). Finally, adherence is an issue in E&G-based interventions: sending emails, handing out flyers or hanging posters does not mean that they are being read, and if they are, it does not mean that their content is understood and applied. This challenge must encourage the realization of clear, pragmatic, and actionable educational content. Examples of educational protocols are shown in Table 1. Formal sessions, visual aids such as flyers and posters and emails are the most commonly used methods for education. If E&G is the only used strategy, it is recommended to expand the range of tools, including flyers, emails and sessions, as well as increase their frequency over time, e.g., with weekly or monthly repetitions, to maximize efficiency.

### Audit and feedback

Audit and/or feedback (A&F) is an effective strategy to reduce inappropriate testing, especially when used in combination with other strategies [67]. The definition of A&F varies, but it typically involves an audit of tests requested, with feedback provided on the tests’ selection practice. A&F can be collective (i.e., anonymous) or individual, the latter being more efficient [34]. Foster et al*.* [34] systematically reviewed A&F-based interventions to improve laboratory test and transfusion ordering in the ICU, regardless of whether the strategy was used alone or integrated with others in a multifaceted study design. They documented that A&F was an efficient strategy to enhance appropriateness of testing, although the overall quality and methodology design was poor. By contrast, in one 81-patient controlled study [68], the impact of an intervention combining feedback (presence of an acute care nurse practitioner during multidisciplinary rounds to discuss next 24 h tests requests) and education (reminders on checklist, reminders on computers and at bedside) did not reach the threshold of statistical significance between intervention and control groups, suggesting that A&F-based interventions may be only moderately effective.

Rachakonda et al*.* [69] combined feedback from clinicians themselves with an educational approach, the latter consisting of monthly formal education on the relevance of testing and pricing information. They achieved a 12% reduction of total costs. The authors measured adherence to feedback by dividing the number of tests authorized the day before (during audit) by the real number of tests effectively requested. Compliance was low (51%), indicating that twice as many tests were requested as the previous day. Compliance to feedback is an interesting parameter to measure, and would be instructive to assess in interventional studies using A&F strategies. Likewise, safety outcomes and effect persistence over time are rarely measured [34], which can nevertheless provide interesting information.

### Gatekeeping

Gatekeeping strategies refer to a constraint on the choice of laboratory tests, usually set by the central (reference) laboratory [11]. This strategy is, for example, used when the laboratory discontinues the possibility of scheduling routine daily tests, and instead imposes lab requests on a test-by-test, day-to-day basis [62].

Few intervention studies used this strategy alone in the ICU (Table 1). In a 48-bed setting, de Bie et al*.* [70] withdrew the daily routine panel (aPTT, INR/PT, blood urea nitrogen [BUN], serum chloride, sodium, albumin, and C-reactive protein [CRP]) and the additional weekly panel (AST, alanine transaminase [ALT], alkaline phosphatase [ALP], amylase, and total bilirubin). They also altered the post-cardiac surgery pre-made panel and the arterial blood gas (ABG) point-of-care testing (POCT) device panels. The total number of tests performed decreased by 24%, whereas the demand rate remained unchanged, thus suggesting that a blood test was indeed indicated in the clinical context, but that one test out of four had previously been inappropriately ordered. The most impacted tests were aPTT, INR/PT, albumin, BUN, serum calcium, chloride, and CRP. The removal of weekly panels had a moderate effect (−18%). Regarding post-cardiac surgery panels, the effect was moderate on creatine kinase isoenzyme MB (−10%) but significant on cardiac troponin (−50%). Finally, the study showed interesting results on ABG stewardship: potassium and glucose were performed in 90% of cases; pH, PO_2_, PCO_2_, hemoglobin and sodium were ordered in only 70–80% of analyses; chloride, ionized calcium and lactate were prescribed in only 30–40% of all ABGs.

Gatekeeping can also take the form of a self-imposed limitation set by the clinicians themselves. In a 191-patient study, Sugarman et al*.* [71] evaluated the adherence to a local standard on seven commonly performed tests (CRP, BUN and electrolytes, serum magnesium, phosphate, liver function tests (LFT), coagulation [not otherwise specified] and FBC), with a self-imposed limit of maximum 25% inappropriate tests. They managed to remain under the 25% limit for CRP, FBC, BUN and creatinine, but exceeded the threshold for LFT (51% of non-indicated tests), magnesium (42%), phosphate (42%) and coagulation tests (40%), ultimately estimating that a quarter of the total costs of the tests were due to inappropriate requests.

Certain gatekeeping principles can help in a more comprehensive strategy. For example, it may be appropriate to define a minimum retesting interval (MRI) for commonly prescribed tests. Tyrrell et al*.* [72] set a 72-h and 24-h MRI on LFT and bone profile respectively, leading to a 23% reduction in tests requested. Prescriptions of bone profile panel dropped by 76% during intervention, whereas prescriptions of calcium and albumin tests increased by 110%, suggesting that clinicians sometimes request an entire panel when only a few tests provide the same clinical information. The authors also compared MRI with a scheduled routine panel testing strategy (i.e., a predefined bundling of tests performed three times a week), along with continuous education and feedback by both clinicians and biochemistry staff. The results showed that scheduled routine panel testing is even more effective than MRI.

### Computerized physician order entry

Interventions to reduce inappropriate testing can focus on computerized physician order entry (CPOE) systems. Reshaping of the electronic request form is a classic intervention that can be coupled with other strategies [73–75]. Alternatively, it takes the form of “prompts” which may appear when selecting a particular test [76–79], choosing a test with MRI [80], or requesting two tests which are redundant in terms of clinical information. Prompts can be set as an indication to the clinician, allowing to override the alert (“soft stop”) with or without needing a written reason for doing so, or can block the test prescription altogether (“hard stop”). Therefore, CPOE prompts can have a gatekeeping component in hard stops or an educational content in soft stops; they can also display indications for testing. For this reason, this category is rather transversal and generally associated with other strategies in MFI [73–80].

Notably, some interventions assessed the effectiveness of CPOE-based strategies alone. In a procalcitonin-specific study, Aloisio et al*.* [81] programmed the CPOE to display a notification when an 80% reduction in initial procalcitonin level had been reached. Procalcitonin is especially used in the ICU for diagnosing severe infection and/or antibiotic stewardship daily, at least until the level is significantly decreases. The authors noted that clinicians tended to mechanically continue testing procalcitonin beyond the threshold of clinically significant variation set at 80% reduction. Automatic notification helped reducing procalcitonin testing by 10%, saving EUR ~ 750 (2019) per bed-year.

CPOE alerts should be used with caution. Repeated alerts may gradually lead clinicians to ignore them, a phenomenon known as “alert fatigue” [82] which often results in alert overriding [83]. Conversely, fear of over-alerting can lead to under-alerting [84]. Therefore, it is important to ensure the right balance when deciding to use these CPOE alerts.

### Multifaceted interventions

Multifaceted interventions (MFI) are studies, where multiples strategies are used concomitantly to manage inappropriate laboratory use. If MFI are considered a category of their own, it is one of the most widely used strategy (Table 1). Several large MFI have been conducted in the ICU, showing strong effectiveness (Table 1). Raad et al*.* [85] led an intervention based on education, gatekeeping and feedback in a 18-bed setting, and observed a one-third reduction of tests over a 9-month period, along with a reduction of POCT testing from 7 to 1 (−83%) test-patient-day and a decrease in the percentage of patients sampled daily from 100 to 12%. This led to an estimated savings of USD 123000 in direct and USD 258000 in indirect costs, with no increase in mortality or length of stay (LOS). Similarly, a study [74] on 3250 patients combined education, guidance, CPOE and feedback-based strategies on routine hematology (FBC), chemistry (BUN and creatinine, electrolytes, magnesium, phosphate, calcium, LFT) and coagulation (INR/PT, aPTT, fibrinogen), and achieved a 28% reduction in test ordered with a sustained 26% reduction over a year, estimating an overall USD 213000 and USD 175000 savings during intervention (6 months) and post-intervention (6 months) periods, respectively. They failed to observe an increase in mortality or LOS, or in morbidity (number of ventilated patients and hemoglobin levels). Merkeley et al*.* [78] designed a 1440-patient study with education on prices, gatekeeping and feedback, demonstrating a total reduction of FBC and electrolytes (not otherwise specified) tests, with a decrease in routine tests (−14% for FBC and -13% for electrolytes) compounded by an increase in non-routine (i.e., punctual) tests (+ 8% for FBC and + 6% for electrolytes), thus suggesting a less frequent use of “ready-made” panels. It led to a CAD 11200 annual saving with no additional adverse outcome. Clouzeau et al*.* [86] conducted a controlled, non-randomized study on 5707 patients (3315 intervention vs. 2392 control) with education, feedback and gatekeeping strategies, achieving a 59% reduction in tests ordered, sustained over a 1-year period, and leading to an annual EUR 500000 cost savings. Recently, Litton et al*.* [87] observed a reduction of 50,000 tests per year with an education, guidance, gatekeeping and feedback-based intervention. They estimated savings up to AUD 800000 per year (30-bed setting) and observed no impact on mortality and LOS. These data suggest that MFI can have lasting effects on the ordering of tests and lead to significant costs savings.

Several interventions are test-specific. Lo et al*.* [76, 77] assessed serum magnesium testing with educational, guidance and CPOE-based interventions. They educated rotating medical and nursing staff in conjunction with a CPOE prompt displaying indications for testing. Non-routine magnesium testing remained stable, while routine testing dropped from 0.71 to 0.57 tests/patient/day (20% decrease) over a 46-week period, with no increase of adverse effects or mortality. Other studies have focused on ABG. Martinez-Balzano et al*.* [88] established local guidance for ABG testing (Table 2) following a literature review, along with educational content (classic educative sessions, posters, stickers on POCT devices, monthly emails), and provided monthly feedback on the intervention. They were able to decrease the ABG performed by 43%. This coincides with another study that coupled education with guidance and reduced inappropriate ABG testing from 54 to 28% [89]. Likewise, a controlled study [90] focused on three common coagulation tests (INR/PT, aPTT and fibrinogen) combining education (face-to-face, posters, emails, prices information) and guidance (via posters), showed that coagulation tests ordering decreased by 64%, whereas control tests only decreased by 15%. The authors did not observe any complications and calculated an approximate AUD ~ 3.8 million (2016) annual economy across Australia and New Zealand. Finally, Viau-Lapointe et al*.* [91] focused on LFT and coagulation testing (not otherwise specified) in a sequential MFI: an audit (interview and online survey) was performed, followed by educational sessions, development of guidance, ending with a gatekeeping strategy on these tests. LFT were reduced from 0.65 to 0.25 (−60%) tests/patient/day, but the reduction in coagulation tests was not statistically significant.

### AI/ML-based assisting tools as future interventions

Recent years have witnessed a growing interest in artificial intelligence and machine learning (AI/ML) algorithms, which are becoming increasingly complex and accurate. There are already various successful examples of AI/ML use in laboratory medicine [92]. Improvement of laboratory testing can be the desired end goal of the algorithm, e.g., when it predicts the amount of information that a test will provide [93], or it is designed to achieve optimization of laboratory resources [94]. Alternatively, improvement of appropriateness can be an indirect consequence, e.g., when the algorithm aim to characterize ICU patients, and that subsequent information on appropriate tests to select can be derived from it [95]. AI/ML models can assist laboratory medicine in achieving appropriateness in multiple ways [96]. For instance, they can predict laboratory test values or identify tests that are likely to give normal results, thus reducing the amount of blood volume. Some models are developed specifically to advise clinicians on which tests to perform, and could thus become a decision-making assisting tool. Models could also be trained on data interpretation to prevent inaccurate interpretation of appropriately prescribed tests, which is a part of the realm of inappropriateness.

Several studies have specifically investigated the use of AI/ML models to limit unnecessary laboratory testing in ICU patients. Cismondi et al*.* [97, 98] applied fuzzy systems algorithms on patients hospitalized in the ICU for gastrointestinal (GI) bleeding with an input of 11 physiological variables (such as heart rate, temperature, oxygen saturation, urine output, etc*.*). They aimed at assessing if eight GI bleeding-related laboratory tests (namely serum calcium, aPTT, PT, hematocrit, fibrinogen, lactate, platelet count and hemoglobin levels) would provide valuable clinical information for decision-making, with the goal of reducing unnecessary tests. The algorithm was able to reduce the tests used by 50% with a false-negative rate of 11.5% (meaning that in 1 case out of 10, the algorithm predicted that the test would yield no information, whereas it would have induced a change in clinicians' decision-making). More recently, Mahani and Pajoohan [99] built an algorithm intended to predict the numeric value of the test requested. They used twelve inputs’ data extracted from the ICU-specific freely available MIMIC-III database [100] including heart and respiratory rates, arterial blood pressure, oxygen saturation, etc*.* Focusing on two laboratory tests (calcium and hematocrit) they used two cohorts of GI bleeding patients (upper versus unspecified) and applied two prediction models (with and without k-means clustering). Prediction error indicator was selected as outcome to better represent effectiveness of prediction models. Calcium had inferior prediction error indicator (~ 9% for upper GI bleeding cohort and ~ 13% for unspecified cohort, respectively) than hematocrit (~ 27% for upper and unspecified GI bleeding cohorts). The model without clustering slightly outperformed the clustering model.

A challenge with prediction algorithms is that they mostly lack dynamicity and adaptability, i.e., they provide a probability for the next test without considering the fact that current decisions will have an impact on future decision-making. In other words, it is particularly important that algorithms consider the fact that a test had previously been omitted because of a certain probability of normality of the test result. To tackle this issue, a team built a deep learning algorithm trained on MIMIC-III database that was at first able to reduce 15% of the twelve most frequently prescribed tests (serum sodium, potassium, chloride, bicarbonate, total calcium, magnesium, phosphate, BUN, creatinine, hemoglobin, platelet count and white blood cells count) at a 5% accuracy cost [101]. They then improved the algorithm by introducing a corruption strategy leading to omission of 20% of laboratory tests requested, while maintaining 98% accuracy in predicting (ab)normal results and transition from normal to abnormal (and vice-versa) [102]. They recently performed an external validation of their algorithm on real-world adult ICU data on the same twelve tests, supporting a possible generalization of their algorithm in the clinical setting [94].

Other approaches have tried to apply information theory’s principles into machine learning algorithms to improve laboratory tests request. An ICU blood draw can yield a large volume of information. The question is whether all this information is clinically relevant, or in other words, whether some of the information in the blood test is redundant, especially over multiple days. Valderrama et al*.* [103] integrated information theory’s concepts of conditional entropy and pretest probability techniques with machine learning to predict whether a test result was likely to be normal or abnormal. They compared the performance of two machine learning algorithms (one with, the second without conditional entropy and pretest probability), showing that the second model had better sensitivity and negative predictive value while being less specific and precise, and that better prediction relies mainly on the pretest probability feature.

Innovative methods involving machine learning are also used to characterize ICU patients. Categorizing patients into subgroups can be useful for predicting outcome or need of intervention, as it can be for selecting laboratory tests. Hyun et al*.* [95] implemented k-means clustering on data from approximately 1500 patients, which included administrative, demographic, medication, and procedural information, in addition to laboratory test data on nine biomarkers (BUN, creatinine, glucose, hemoglobin, platelet count, red and white blood cells count, serum sodium and potassium). They found that three was the optimal number of clusters, with significant difference in mortality and morbidity (intubation, cardiac medications and blood administration during ICU stay). They also identified three tests of particular interest for discriminating patients’ outcomes, namely creatinine, BUN and potassium, the values of which were significantly increased in the higher mortality cluster. This suggests that patients clustering could lead to personalized clinical pathways, and thereby identify tests to be performed or avoided in specific subgroups.

## Discussion

This review addresses five intervention categories aimed at enhancing the appropriateness of laboratory testing in the ICU. We include a sixth category exploring the potential of AI in such interventions. Overall, the interventions proved to be effective, as they resulted in a reduction in tests of approximately 30%, depending on the type of intervention, methodology, setting, and tests studied (Table 1). This coincides with the estimated 20–40% of inappropriate tests reported in the literature [8]. The most prevalent categories are MFI and E&G-based interventions (Table 1), in line with other non-ICU-specific reviews [20, 23].

**Fig. 1:**
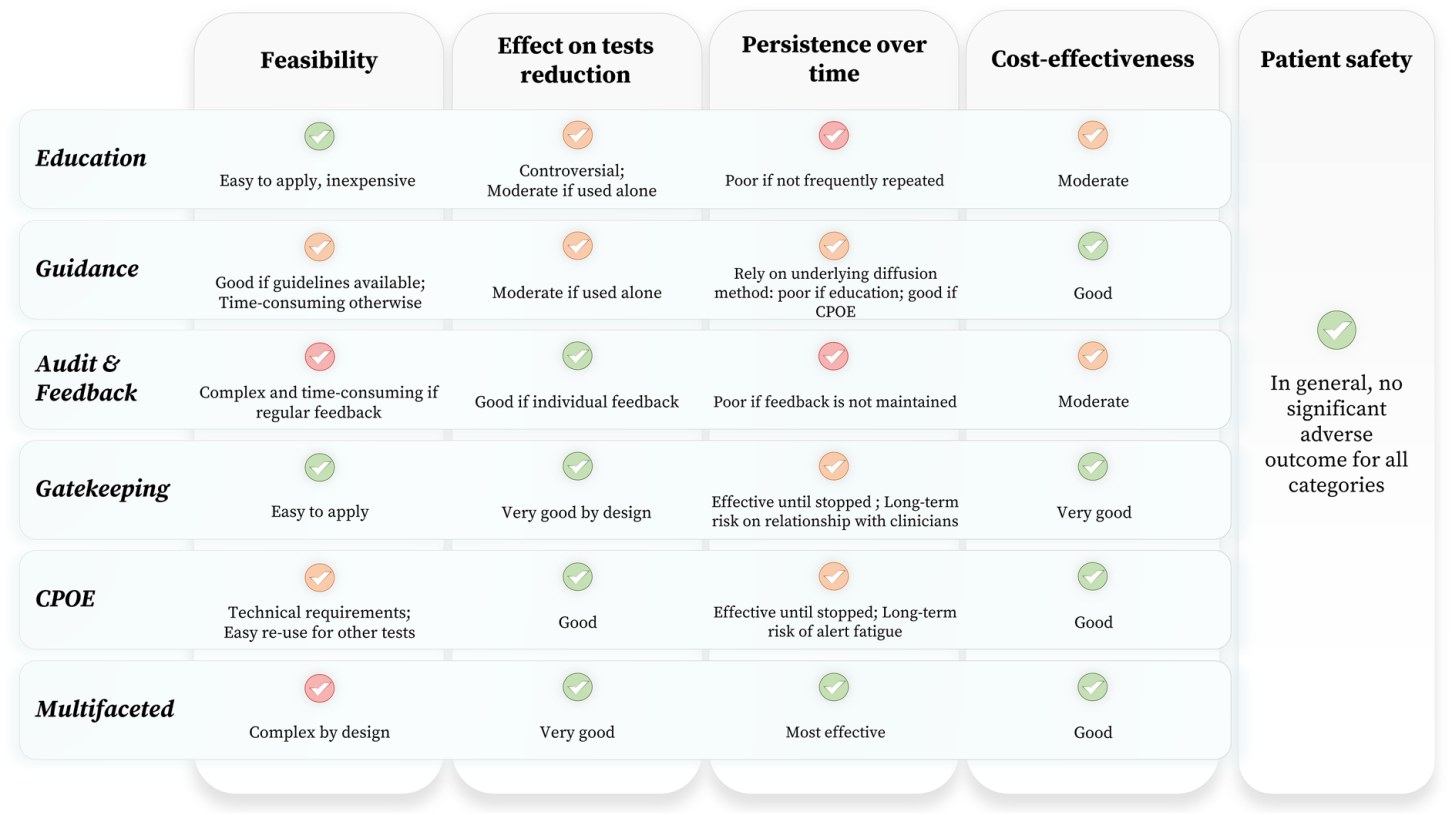
Qualitative comparison of interventions to improve the appropriateness of laboratory testing in the ICU. Comparison is given for education, guidance, audit & feedback, gatekeeping, computerized physician order entry (CPOE) and multifaceted interventions in terms of feasibility, effectiveness, persistence over time (sustainability), cost-effectiveness, and patient safety. AI-based interventions are not represented

Each strategy has relative benefits and drawbacks (Fig. 1). Education is an accessible and inexpensive approach to elicit a test reduction behavior. However, it requires an effortful and consistent application to effect a notable change in prescribing behavior. There is variation in reported efficacy of education-based interventions in the literature [3, 4, 21, 32, 33]. We found a good effectiveness of E&G-based interventions with low persistence of effect over time if the intervention is not re-enforced (Table 1). These observations are consistent with those of a systematic review [30] on interventions conducted among primary care physicians. Possible solutions include continuous training for rotating staff (e.g., residents) and displaying costs of laboratory tests [4, 11, 32, 104]. Providing indications for testing is an often used and effective strategy. In the unique context of ICU, it is challenging to establish one-fit-all guidance because of the wide disparity of complex clinical conditions. Therefore, indications for testing frequently differ between countries, or even locally, among hospitals [64, 66, 105]. Yet, implementing locally established guidance alone seems not sufficient to overcome the problem of inappropriateness [106]. Moreover, this heterogeneity complicates the generalization of results of guidance-based interventions. There is evidence that adherence to guidelines is suboptimal [107], which may lead to undesirable outcomes [108]. Several barriers to guideline adherence have been identified, namely awareness of these guidelines, familiarity and agreement with their content, resistance to change (“normal practice inertia”), external barriers (equipment, financial resources), conflicts between guidelines, or simply because they do not adequately reflect real-world situations [107, 108]. Finally, guidance may be subject to bias [109]. A&F is an effective strategy, but it tends to be more effective when individual feedback is provided. Compliance to A&F could prove to be an important determinant of success, and should be ideally assessed. Furthermore, providing regular and consistent feedback is complex and time-consuming [33]. Gatekeeping is among the most effective strategies. However, in the long term, it can impair the relationship between the laboratory and the clinicians. Collaboration with clinicians (e.g., via education and bilateral good practice standards establishment) should prevail over an unilateral stewardship from the central laboratory [106]. Gatekeeping can be integrated into a broader policy, e.g., by implementing MRI or scheduled routine panel testing in consultation with clinicians, or to limit particular tests to certain wards [11]. Collaboration with care staff is an important element for gatekeeping strategies’ long-term success. CPOE-based strategies have proven to be successful and can be used either alone (e.g., modification of the ordering form) or as a support for other types of interventions (e.g., education or gatekeeping). Caution should be taken when using alert systems to find the optimum alert level, in order to prevent alert fatigue [25, 83, 84]. MFI appear to be the most used and effective strategy to reduce inappropriateness of laboratory requests (Table 1), as already reported [20, 21, 23, 30, 39]. They lead to significant costs savings and show the higher persistence of effect over time (Table 1). Nonetheless, many MFI focused on a single analyte or type of tests. It would be worthwhile to conduct rigorous multifaceted studies on large panels of tests. Few studies have evaluated the long-term effectiveness of interventions. Only 10 out of the 45 studies retrieved addressed the persistent effect of intervention at 1 year, only 2 beyond 1 year (Table 1). Therefore, further studies are needed to evaluate the persistence of long-term effects of intervention.

In the near future, AI/ML-based assisting tools will probably be an important ally for laboratory medicine [36–38]. It could be applied to enhancement of appropriate testing in various ways. By predicting the amount of information that the repetition of a test provide, ‘AI/ML-based MRI’ could be considered. Regarding commonly prescribed pairs (e.g., sodium/chloride), we could think of performing only one of the two tests (e.g., sodium) and predicting the result of the second (in our example, chloride) with AI/ML prediction models. AI-based clustering of patients could be another way of improving appropriateness of laboratory testing, by defining the most relevant tests to select for each phenotype. If so, AI/ML-based tools will have to be compliant with the European in vitro diagnostic medical devices regulation (IVDR) [110]. For the moment, this poses several challenges, the most critical of which being interpretability and transparency due to the inherent “black box” design of AI tools [111, 112]. AI/ML algorithms pose other challenges for the future of laboratory medicine in both technical and ethical perspectives. As Pennestri and Banfi [113] state, “The performance of AI technologies highly depends on the quality of inputs, the context in which they are collected and the way they are interpreted”. For example, an AI/ML model may produce biased output due to input data [114]. The use of AI/ML models also raises the question of responsibility when a necessary test is not performed due to the model's failure to recommend it, potentially jeopardizing patient safety [115]. Some authors have also expressed concern about whether or not to inform the patient that a decision was based on AI/ML suggestions [36, 116]. Pennestri and Banfi also highlight a subtle ethical challenge of AI/ML implementation regarding patient autonomy, as AI/ML models are not currently taking patient preference into account: the test that the patient *need* the most may not necessarily reflect what patient *prefers* [113]. In addition, the use of AI/ML models raises concerns about the acquisition and safe storage of big data, both in technical, financial, and ethical terms [36]. Currently, the implementation of AI/ML models in healthcare still faces a major challenge due to the doubtful inclination or even rejection from healthcare professionals [116]. This seems mainly due to concerns about job security and quality of care after AI implementation [117]. At present, it is unlikely that AI will replace specialists in laboratory medicine in many laboratory processes. Evidence shows that combined human/AI processes in detection of breast cancer cells are more efficient than human pathologists- or AI-alone processes, respectively [4, 118]. This synergy effect suggests that AI-based tools would for now be *assisting* ones.

An important aspect of appropriate testing improvement is to safeguard patient safety. On the one hand overuse should be minimized without omitting tests important for the clinical management. On the other hand, underuse should drive the necessary tests to be performed without requesting additional inappropriate tests. Achieving the optimal balance is inherently challenging. Nevertheless, no successful intervention that assessed safety outcomes in our review has led to a deterioration in patient safety (Table 1). Although we cannot exclude publication bias, it is a strong argument in advocating for inappropriate tests reduction.

A major challenge in interpreting data from these interventions is the overall poor quality of design, lack of standardization in methodology and diversity of outcomes [23, 27, 30, 104]. Table 3 summarizes various confounding factors that explain this heterogeneity. For example, studies have shown that size (reflected by the number of involved healthcare workers) is a significant confounding factor for the effectiveness of an intervention [119]. Other confounding effects may include geographical location, local culture about appropriateness of laboratory use, or teaching versus non-teaching status of the hospital. The International Federation of Clinical Chemistry and Laboratory Medicine (IFCC) and its European counterpart (EFLM) have put great effort towards the standardization process [120, 121]. For example, the EFLM is working on harmonization of MRI across European countries [122]. However, there is no clear standardization on conducting and reporting interventions to reduce inappropriate laboratory testing. Standardization of the methodology design of interventions would benefit to the more efficient generalization of data collected.

**Table 3:** Factors of heterogeneity in interventions to improve laboratory testing in the intensive care unit

Methodological
Tests selected for the study Type of intervention conducted Single strategy vs. multifaceted intervention Duration of intervention protocol Outcome(s) measured Rate of outcome(s) assessment Last measurement carried out over time
Organizational
Hospital size Hospital internal organization Local testing protocols or guidance Local availability of tests Local knowledge and culture of appropriateness of laboratory testing Teaching vs. non-teaching status
Systemic
Currency differences between countries National organization of healthcare system Public–private balance Organization of insurance and reimbursement systems

Three main categories regroup the factors that explain the heterogeneity of methods and the difficulty of generalizing data in interventions aimed at improving laboratory testing

The definition of inappropriateness varies across studies [34], each with its own strengths and weaknesses. Nevertheless, it is crucial to define whether a laboratory test is appropriate or not, because it determines the outcome measured in the study. According to some authors, a laboratory test is considered inappropriate when it has no meaningful impact on therapy or yields a normal result [123]. However, this definition may not always apply, especially in conditions such as the acute coronary syndrome, where a negative cardiac troponin level is a significant finding. In comparison, Lundberg suggested that an intervention is inappropriate when harm outweighs benefits [124]. Appropriateness has also been associated with adherence to organizational guidelines [106, 125] or self-referral [126]. Often, appropriateness is defined using literature or expert opinion [125]. Some authors have suggested to refine it by distinguishing between inappropriate *requests* (the question asked is clinically inappropriate), inappropriate *tests* (the question asked is clinically relevant, but the wrong test is selected by the clinician, or the wrong test is performed by the laboratory) and *unnecessary requests* (the question asked may have been clinically appropriate, but may no longer be so at the time of testing) [33]. The value-based healthcare (VBHC) approach offers a more objective definition of appropriateness. VBHC is focused on determining the value that an intervention provides, which means evaluating the outcomes achieved per money spent [127]. A test can be considered inappropriate if it is of low value. According to Colla et al*.* [67], low value care refers to application of care that is unlikely to benefit the patient considering the cost, alternative options available, and patient preferences. To determine the appropriateness of a test, we emphasize the need for comprehensive evaluation of the clinical utility of prescribing the test in conjunction with physiological or pharmacological principles (e.g., half-life) of the molecular target of the test.

Test ordering decision-making is a complex task that requires time and high-intensity attention. When interviewed, ICU physicians disclose that they do not have the necessary time to thoroughly assess the appropriate tests to order from the unnecessary ones [84]. In this context, interventions to reduce inappropriateness can be perceived as an additional strain. On the contrary, well-executed interventions can influence physicians’ test-ordering behavior [22]. For example, by making appropriate tests easier and inappropriate tests more difficult to select, guidance- and CPOE-based interventions favor efficiency. In contrast, education, financial incentives, and A&F interventions favor thoroughness. It also highlights the role of laboratory staff in the pursuit of appropriateness. As physicians have little time to devote to the proper utilization of laboratory resources, specialists in laboratory medicine should intensify the collaboration to reduce inappropriate testing and to proactively become “knowledge manager[s]” [128]. Specialists in laboratory medicine have the responsibility to ensure communication with users in order to provide education on latest evidence for tests selection and advice on appropriate interpretation of tests [129–132].

Inappropriate laboratory testing concerns over—as much as underuse. It seems that underuse is even twice as frequent as its counterpart [8]. However, there is a bias toward the reduction of overuse in the literature of interventions aimed at improving testing appropriateness. This may be because of the easier assessment of tests reduction and direct costs savings in reduction of overuse. In the interventions we reviewed, only one [84] mentioned underuse. Notably, it was not in accordance with Zhi et al*.*’s estimations [8], as the results showed an overuse of procalcitonin in one out of five tests, whereas underuse was estimated to occur in one out of 38 tests [84]. It is likely that underuse vary depending on the test concerned [133–135]. Future studies assessing consequences of reducing underuse are needed.

Several limitations of this review deserve mention. Although comprehensive, our literature search was not systematic. We did not systematically evaluate the quality of studies, or their potential biases. Yet, a 2015 systematic review emphasizes the poor quality of interventional studies in the general setting [23]. Our study found an overall poor quality of methodology and reporting. Forty-one percent (18 out of 44) of the studies we retrieved were conference abstracts or letters to editors that often lacked full details of the methodology used. This study may therefore have limitations in terms of its breadth, depth, and comprehensiveness. We decided to include conference abstracts to increase comprehensiveness. As discussed above, the lack of standardization in study design complicates data generalization, and we cannot exclude the presence of publication bias. Caution is thus advised with certain numbers, particularly with costs savings. Nevertheless, most of the numbers are estimates, and the central message remains the trend towards reduction in the number of inappropriate tests, and potential savings made, while preserving patient safety. Delimiting studies into categories can introduce bias, and this division can appear artificial for certain studies that do not clearly fall into one category or another. We had to balance between facilitating understanding through a more general classification and the rigor of a more specific but numerous classifications. However, we classified our data according to literature standards as closely as possible [4, 11, 32, 33, 39]. Our review focused on ICU adult patients. We did not investigate microbiology, in that it is a highly specific diagnostic area, with its own methods, tests and body of literature.

**Fig. 2:**
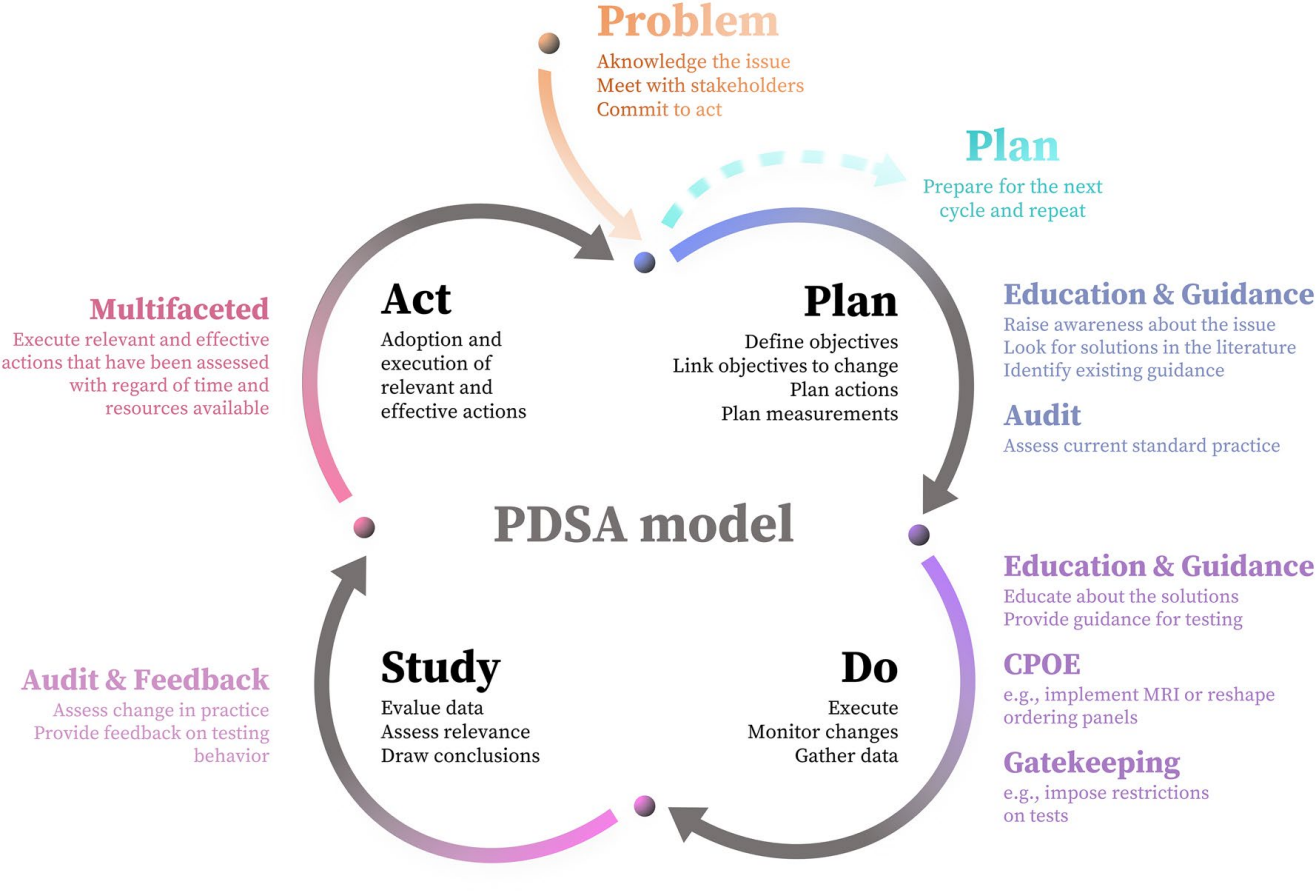
Schematic representation of the Plan-Do-Study-Act (PDSA) model. In the center of the figure, the objectives of the four stages of the PDSA model are summarized. At the periphery are examples of possible types of interventions for each stage

The plan–do–study–act (PDSA) cycle is a frequently used model for improving process and practice, such as reducing inappropriate use of laboratory resources. The first stage involves defining objectives, linking them to desired changes, determining necessary actions to bring about change, and planning how to measure the success of the change. In the second stage, the planned actions are performed and data is collected. During the third stage, the effectiveness of the actions is evaluated, and their relevance to the desired objective is assessed. In the fourth stage, the data analyzed in the previous stage is used to determine whether the change can be adopted and to plan the next PDSA cycle. In a broader sense, the PDSA model provides an analogy to describe the process involved in an intervention to limit inappropriate use of laboratory resources, and can be used to develop effective and sustainable interventions (Fig. 2). Based on our literature review, we recommend utilizing a multi-strategy approach. The first step towards improvement is recognizing a problem or an area for improvement and committing to acting. We suggest starting any intervention by defining the desired objective and possible options, as well as meeting with stakeholders regarding the objectives. The initial logical step of an intervention would be to explicitly state the need for change by explaining the problem, the reasons for change, and the available solutions through stakeholder education. We advise educating about the problem (e.g., raising awareness about inappropriateness), as well as the selected solutions to face it (e.g., the strategies that will be used to address the problem). Conducting a literature review can provide objective facts to support problem definition, evaluate existing solutions from the literature, and identify available guidance (see Table 2). At this stage, an audit can evaluate current standard practices. From there actions can be taken, which may include the various strategies discussed such as implementing MRI or reshaping ordering forms or panels (i.e., CPOE), and imposing new restrictions on certain tests (i.e., gatekeeping) (Fig. 1). It is important to evaluate the impact of initial strategies and make the necessary changes. An audit and feedback strategy can be used to assess the change brought about by the intervention compared to the pre-intervention situation. Although this strategy may be complex and time-consuming, it is an effective way to assess progress and make necessary corrections. The audit results can determine whether to maintain current actions or adapt the intervention. If necessary, the education and guidance cycle can be renewed to increase effectiveness. The cycle can be repeated until the desired outcome is achieved, or even indefinitely. Few studies have assessed the effectiveness of interventions beyond 1 year. Therefore, we suggest frequent renewal of PDSA cycles and/or long-term evaluation of intervention effectiveness. Throughout the entire process, AI/ML models can assist in selecting, implementing, or optimizing strategies, and even provide additional support through future applications. Finally, we emphasize that maintaining ongoing communication with clinicians and other stakeholders throughout the entire process is key to ensure successful implementation of the changes. We believe that this framework can lead to interventions that maximize effectiveness in reducing inappropriate use of laboratory tests.

## Conclusions

We reviewed interventions aimed at improving appropriate laboratory resources utilization in the ICU. We identified six discrete categories of interventions: education and guidance (E&G)e, audit and feedback (A&F), gatekeeping, computerized physician order entry, multifaceted and AI/ML-based interventions. We provided an assessment of respective benefits and drawbacks. The most represented categories of interventions are E&G-based and MFI. The most efficient and long-lasting interventions are MFI. AI/ML-based assisting tools interventions could be promising for enhancing the appropriate of testing in the future. Collaboration between clinicians and laboratory staff is key to improve rational laboratory utilization. Reduction of overuse is overrepresented in the literature in comparison to improvement of underuse. Moreover, overall methodological quality is poor and study designs lack standardization. Further studies on underuse of laboratory testing in the ICU as well as standardization of methodology for interventions are needed. We provide practical guidance for optimizing the effectiveness of an intervention protocol designed to limit inappropriate use of laboratory resources.

### Supplementary Information

**Additional file 1: Table S1**. Methodological details of the review.

## Data Availability

The data extracted, analyzed, and presented in Tables 2 and 3 are available from the corresponding author upon request.

## Abbreviations

A&F: Audit and feedback
ABG: Arterial blood gas
AI/ML: Artificial intelligence and machine learning
ALP: Alkaline phosphatase
ALT: Aspartate aminotransferase
aPTT: Activated partial thromboplastin time
AST: Alanine transaminase
AUD: Australian dollar
BMP: Basic metabolic panel
BUN: Blood urea nitrogen
CAD: Canadian dollar
CPOE: Computerized physician order entry
CRP: C-reactive protein
EUR: Euro
FBC: Full blood count
GI: Gastrointestinal
INR: International normalized ratio
ICU: Intensive care unit
LFT: Liver function tests
LOS: Length of stay
MFI: Multifaceted intervention
MRI: Minimum retesting interval
POCT: Point-of-care testing
PT: Prothrombin time
USD: US dollar
