# Supplementary material for: Interventions to improve appropriateness of laboratory testing in the intensive care unit: a narrative review
Source: Ann Intensive Care. 2024 Jan 15;14:9. doi: 10.1186/s13613-024-01244-y (PMC10789714; doi:10.1186/s13613-024-01244-y)
Supplement: Supplementary file 1 — Additional file 1: Table S1. Methodological details of the review. [file 13613_2024_1244_MOESM1_ESM.docx]

# Additional file 1.

**METHODOLOGICAL DETAILS OF THE REVIEW.**

## Search strategy

We searched literature from 2008 to 2023 in PubMed, Embase, Scopus, and Google Scholar databases between April and June 2023 (last access: 30/06/2023). We used keywords related to laboratory medicine, appropriateness, demand management, and ICU, using an appropriate search strategy [1] (**Table 1**). We selected articles with the following criteria: ICU-specificity; adult patients; biochemistry or hematology tests; interventional studies. We selected papers from non-ICU departments to compare trends found in the ICU. References of articles retrieved by our search methodology were also scanned. We scanned the references provided by PubMed as similar to the references found by our search strategy. Additional punctual searches were performed on particular topics. We did not systematically assess the quality of the studies and potential biases.

Table 1.

Keywords used for literature searching strategy.

| **Intensive care unit** | **Laboratory utilization** | **Demand management strategy** | **Appropriateness** | **MeSH Terms (for PubMed)** |
| --- | --- | --- | --- | --- |
| "intensive care unit*"  ICU  "critical care"  "critically ill"  "critical patient" | "laboratory test*"  "laboratory request*"  "laboratory order*"  "test* request*"  labs  prescription  "laboratory utilization"  "laboratory use"  request* | "minimum retesting interval*"  "retesting interval*"  MRI  "demand management"  "demand management strateg*"  manag*  DMS  "test* repetition"  "repetitive test*"  frequency filtering | decreas*  improv*  enhance*  unnecessary  inappropriate*  appropriate*  relevance  reduc*  overuse  overutilization  underuse  underutilization  optimiz*  optimis*  redundan*  needless | Clinical Laboratory Techniques  Clinical Laboratory Services  Critical Care  Intensive Care Units  Procedures and Techniques Utilization  Cost-Benefice Analysis  Unnecessary Procedures  Medical Overuse |

## Categorization

We grouped interventions having mechanisms in common into six discrete categories, identified according to the strategies detailed in non-ICU-specific literature [2-10]: education and guidance (E&G), audit and feedback (A&F), gatekeeping, computerized physician order entry (CPOE) solutions, multifaceted interventions (MFI) and artificial intelligence and machine learning (AI/ML)-based solutions. “Education” refers to any type of learning content and includes reminders, while “guidance” regroups any type of indications on which test to perform, whether it refers to (inter)national standards (guidelines *per se*) or locally established guidance. Since they are often closely related, for consistency with the literature, we have decided to group them in the same category. “Audit and feedback” encompasses strategies that evaluate tests requested or provide feedback, including advice on tests selection. “Gatekeeping” refers to any kind of constraint-based solution that limits test ordering. CPOE-based solutions are a cross-cutting category of strategies using the CPOE, whether alone (e.g., modification of the ordering form) or as a support for other types of interventions (e.g., education or gatekeeping), and includes electronic prompts (“pop-ups”). Most modern laboratories rely on a laboratory information system coupled with the hospital information system, and test ordering is mainly done through CPOE. Therefore, we have decided to integrate the reshaping of ordering forms and/or laboratory ordering profiles into the CPOE category. MFI covers all interventions that used multiple strategies concomitantly. Given recent spotlight on AI for laboratory medicine [2], a seventh category includes interventions using deep learning or other algorithm-rooted systems to improve appropriate laboratory testing.

## Economic data

The original economic data (extracted from the original publication) are given in currency from the original article according to ISO4217:2015 standards [11], without adjustment for inflation. Currencies used in this review include US dollar (USD), Australian dollar (AUD), Canadian dollar (CAD) and Euro (EUR).

## References

1. Bramer WM, de Jonge GB, Rethlefsen ML, Mast F, Kleijnen J. A systematic approach to searching: an efficient and complete method to develop literature searches. J Med Libr Assoc. 2018;106(4):531-41.

2. Mrazek C, Haschke-Becher E, Felder TK, Keppel MH, Oberkofler H, Cadamuro J. Laboratory Demand Management Strategies-An Overview. Diagnostics (Basel). 2021;11(7).

3. Panteghini M, Dolci A, Birindelli S, Szoke D, Aloisio E, Caruso S. Pursuing appropriateness of laboratory tests: a 15-year experience in an academic medical institution. Clin Chem Lab Med. 2022;60(11):1706-18.

4. Ferraro S, Panteghini M. The role of laboratory in ensuring appropriate test requests. Clin Biochem. 2017;50(10-11):555-61.

5. Freedman DB. Towards Better Test Utilization - Strategies to Improve Physician Ordering and Their Impact on Patient Outcomes. EJIFCC. 2015;26(1):15-30.

6. Alonso-Cerezo MC, Martín JS, García Montes MA, de la Iglesia VM. Appropriate utilization of clinical laboratory tests. Clin Chem Lab Med. 2009;47(12):1461-5.

7. Fraser CG, Woodford FP. Strategies to modify the test-requesting patterns of clinicians. Ann Clin Biochem. 1987;24 ( Pt 3):223-31.

8. Huck A, Lewandrowski K. Utilization management in the clinical laboratory: an introduction and overview of the literature. Clin Chim Acta. 2014;427:111-7.

9. Smellie WS. Demand management and test request rationalization. Ann Clin Biochem. 2012;49(Pt 4):323-36.

10. Cadogan SL, Browne JP, Bradley CP, Cahill MR. The effectiveness of interventions to improve laboratory requesting patterns among primary care physicians: a systematic review. Implement Sci. 2015;10(1).

11. International Organization for Standardization (ISO). ISO4217:2015: Codes for the representation of currencies 2015 [Available from: <https://www.iso.org/standard/64758.html>].
